# Molecular evidence for hybridization in *Colias* (Lepidoptera: Pieridae): are *Colias* hybrids really hybrids?

**DOI:** 10.1002/ece3.1574

**Published:** 2015-06-25

**Authors:** Heather E Dwyer, Marie Jasieniuk, Miki Okada, Arthur M Shapiro

**Affiliations:** 1 Eberly Center for Teaching Excellence and Educational Innovation, Carnegie Mellon UniversityPittsburgh, Pennsylvania, 15213; 2 Department of Plant Sciences, University of CaliforniaDavis, California, 95616; 3 Department of Evolution and Ecology, University of CaliforniaDavis, California, 95616

**Keywords:** Amplified fragment length polymorphism, *Colias eriphyle*, *Colias eurytheme*, hybridization, population structure

## Abstract

Gene flow and hybridization among species dramatically affect our understanding of the species as a biological unit, species relationships, and species adaptations. In North American *Colias eurytheme* and *Colias eriphyle,* there has been historical debate over the extent of hybridization occurring and the identity of phenotypically intermediate individuals as genetic hybrids. This study assesses the population structure of these two species to measure the extent of hybridization and the genetic identity of phenotypic intermediates as hybrids. Amplified fragment length polymorphism (AFLP) marker analysis was performed on 378 specimens collected from northern California and Nevada. Population structure was inferred using a Bayesian/Markov chain Monte Carlo method, which probabilistically assigns individuals to genetic clusters. Three genetic clusters provided the best fit for the data. *C. eurytheme* individuals were primarily assigned to two closely related clusters, and *C. eriphyle* individuals were mostly assigned to a third, more distantly related cluster. There appeared to be significant hybridization between the two species. Individuals of intermediate phenotype (putative hybrids) were found to be genetically indistinguishable from *C. eriphyle*, indicating that previous work based on the assumption that these intermediate forms are hybrids may warrant reconsideration.

## Introduction

**Figure 1:**
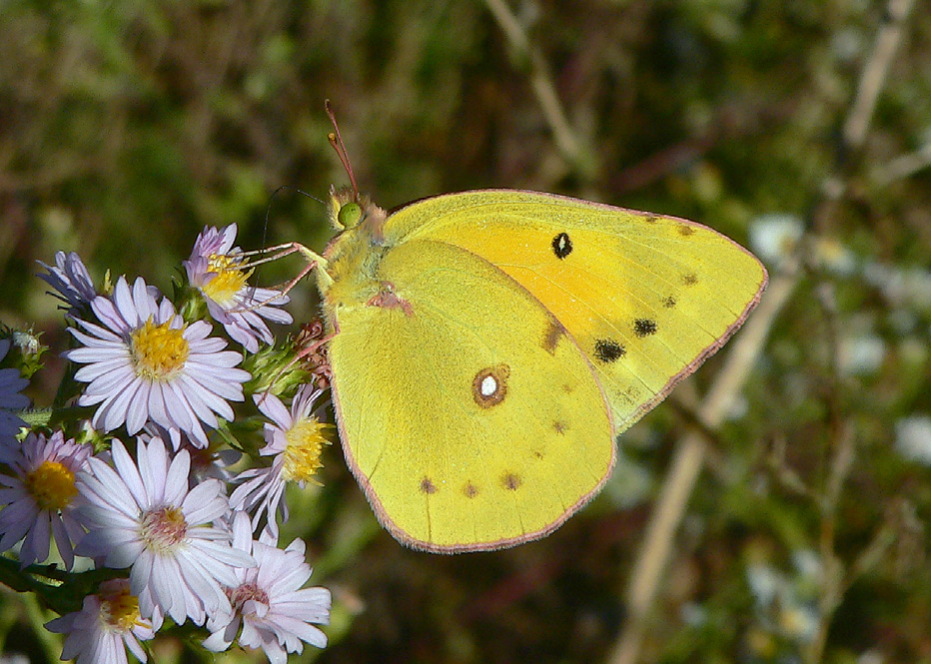
*Colias eurytheme,* ventral side. Photograph credit: Greg Hume.

Detection of gene flow between populations is a critical component of understanding a species’ population biology. At a higher taxonomic level, interspecific gene flow can lead to sustained hybridization and introgression. A better understanding of the levels of admixture and hybridization between species allows us to consider both the evolutionary relatedness of different species, and the potential for interbred species to respond to changing environmental pressures via hybrid swarms or increased selection for individuals of mixed ancestry. In North American butterflies species *Colias eurytheme* (Fig.1) and *Colias eriphyle,* there has been historical debate over the extent of hybridization occurring and the identity of phenotypically intermediate individuals as genetic hybrids. This study assesses the population structure of these two species to measure the extent of hybridization and the genetic identity of phenotypic intermediates as hybrids.

Hybridization in the sulfur butterfly complex (Pieridae: Coliadinae), which includes *Colias eurytheme* (orange sulfur), *Colias philodice* (clouded sulfur), and *Colias eriphyle* (yellow sulfur), has long been recognized (Gerould 1943; Ae 1959; Taylor 1972). The status of *C. eurytheme* and *C. eriphyle* as distinct species has been controversial for well over a century (Gerould 1946). *C. eurytheme* is ubiquitous across North America, its range spanning most of the continental United States and Mexico. *C. eriphyle,* once considered the same species as its close relative *C. philodice,* occupies much of the western United States and British Columbia. Currently, *C. eurytheme* and *C. eriphyle* are understood to be sister taxa; *Colias philodice* (the clouded sulfur) is the sister group to these two species (Pollock et al. 1998; Wheat and Watt 2008). *C. eurytheme* and *C. eriphyle* have been postulated to hybridize, as phenotypic intermediates of *C. eurytheme* and *C. eriphyle* have been observed in the western United States in areas of sympatry, including the eastern side of the Sierra Nevada mountain range (Hovanitz 1943; Gerould 1946; Clark and Clark 1951).

In general, when previously isolated populations become sympatric and hybridize, one of two possible patterns typically occurs: (1) genetic incompatibility results in selection for characters that reduce interspecific crosses ultimately leading to complete reproductive isolation, or (2) lack of incompatibility and divergence leads to an increase in hybridization rates (Taylor 1972). The *Colias* system, however, presents an unusual pattern. The observed frequencies of phenotypic intermediates suggest that the two species experience low but consistent levels of gene flow wherever they occur in sympatry, while the two parent species retain distinct identities (Clark and Clark 1951; Gerould 1946; Hovanitz 1943; Taylor 1972; A. M. Shapiro, unpubl. data). Following the initial occurrence of species range overlap in the 1920s and 1930s, prevalence of these intermediate individuals briefly peaked and then equilibrated around 10–15% (Clark 1932; Gerould 1946; Clark and Clark 1951; Taylor 1972; Jahner et al. 2012), although some localities in Siskiyou County, California, appear to retain unusually high levels of phenotypic intermediates (A. M. Shapiro, unpubl. data). These hybrid frequency data are based on observations of individuals of intermediate coloration; however, no prior genetic analysis has been performed to confirm that these individuals are in fact of mixed parentage. Moreover, there is yet no information regarding genetic evidence for the extent of hybridization occurring between the two species. A better understanding of the population genetic structure of these species would allow stronger inferences about the evolutionary history of these groups and elucidate the levels of hybridization occurring between *C. eurytheme* and *C. eriphyle*.

*Colias* butterflies provide a convenient system in which to study hybridization. *C. eurytheme* and *C. eriphyle* are present at low and high elevations in the western United States and across the Sierra Nevada (Hovanitz 1943; Gerould 1946; Clark and Clark 1951). Both species are considered to be ruderal with high dispersal ability, which has historically led to gene flow, introgression, and hybridization. Specifically, *C. eurytheme* underwent a rapid range expansion in the 1930s, causing range overlap between the two species and immediate documented hybridization (Clark 1932; Gerould 1946; Clark and Clark 1951).

Various molecular genetic tools and methods have been used to measure hybridization and introgression in both plants and animals. In butterflies, amplified fragment length polymorphism (AFLP) markers have been used to measure hybridization and produce linkage maps of hybridizing butterfly species successfully (Wang and Porter 2004; Kronforst et al. 2006; Kronforst 2008; Winter and Porter 2010). Investigations of population structure allow inferences concerning the presence and relatedness of genetic groups (i.e. populations) of individuals and the extent of interindividual, interpopulation, and interspecies gene flow (admixture) occurring, and thus whether hybridization is occurring between species.

Using AFLP marker techniques, this research addressed the following questions: (1) Is there genetic evidence for introgression between *C. eurytheme* and *C. eriphyle*, and if so, what is the extent of that introgression? (2) Is there genetic evidence that individuals of intermediate coloration are in fact of mixed parentage (i.e., hybrid or back-crossed individuals)? (3) Is there genetic evidence of higher levels of introgression at a site where high frequencies of phenotypic intermediates have been historically observed?

## Materials and Methods

### Study system

*Colias eurytheme* is extremely common and has a wide range across the United States, excepting the northernmost regions and peninsular Florida (Gerould 1943; Hovanitz 1943, 1950). In California, it occurs in almost any open habitat and feeds on agricultural alfalfa and other legumes. Adult color morphology is variable due to genetics and environment (season), with the ground color of the dorsal side of the forewings ranging from lighter yellow with a fairly distinct orange blotch (early spring forms) to saturated orange forewings (summer) (Gerould 1943). Males have solid black wing borders while female wing borders are black with ground color (orange to yellow) spots.

*Colias eriphyle* is a close relative of *C. eurytheme* and feeds on the same host plants (Wheat and Watt 2008). Its range overlaps that of *C. eurytheme* east of the Sierran crest, but it does not occur in Mediterranean California. It is most easily distinguished from *C. eurytheme* by its solid, pale yellow dorsal wing ground color (Taylor 1972). *C. eriphyle* undergoes three generations at high elevations of the Sierra Nevada (Jahner et al. 2012). *C. eriphyle* has historically been considered a subspecies of *Colias philodice*, another close relative of *C. eurytheme* found throughout the eastern United States, and is often referred to as *C. philodice* in the literature (Wheat and Watt 2008).

The phylogenetic relationships among *C. eurytheme, C. eriphyle,* and *C. philodice* are poorly understood. The species have undergone parallel evolution, and their traits are similar in many respects (Porter and Levin 2007). Putative hybrids occur between *C. eurytheme* and *C. philodice* in the eastern United States (Gerould 1946) and between *C. eurytheme* and *C. eriphyle* in the west (Taylor 1972). A few phylogenetic analyses have been conducted in this system. Pollock et al. (1998) used mtDNA to confirm *C. eurytheme* and *C. philodice eriphyle* as close relatives; since then, Wheat and Watt (2008) constructed mtDNA-based phylogenies showing *C. eurytheme* and *C. eriphyle* as sister taxa (i.e., they are more closely related to each other than to *C. philodice*). Wang and Porter (2004) used amplified fragment length polymorphism (AFLP) to generate a linkage map of interbreeding *C. eurytheme* and *C. philodice,* which serves as a starting point for further genetic analyses of introgression and hybridization between these two species.

**Figure 2:**
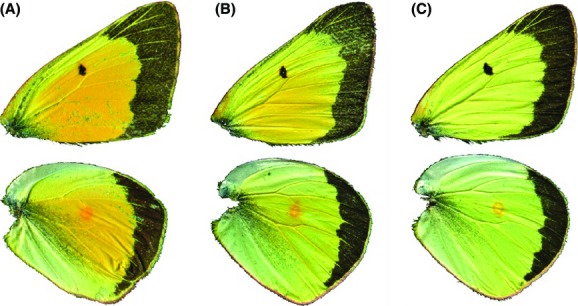
Phenotypes of *Colias*. (A) Dorsal surface of *Colias eurytheme*. (B) Dorsal surface of a phenotypically intermediate individual (putative hybrid). (C) Dorsal surface of *Colias eriphyle*.

On the eastern side of the Sierra Nevada, *C. eurytheme* and *C. eriphyle* occur in sympatry and, in accordance with the presence of phenotypic intermediates, appear to hybridize. The phenotypically intermediate putative hybrids display an even, intermediate light orange dorsal wing color (Gerould 1943) (Fig.2). Although no molecular analysis confirming hybrids has been performed, putative hybrids identified on the basis of intermediate phenotype have been observed to comprise up to 15% of the mixed population (Hovanitz 1943; Gerould 1946; Taylor 1972; Jahner et al. 2012). Most putative hybrids are observed to be males, in accordance with Haldane’s rule (Haldane 1922), as Lepidoptera follow the ZW system (in which females are the heterogametic sex) (Presgraves and Orr 1998). The putative hybrid phenotypes are unknown in California west of the Sierra Nevada crest, where only *C. eurytheme* occurs. There are no known populations of *C. eriphyle* in California or Nevada where *C. eurytheme* does not occur. However, the status of these phenotypic intermediates as true hybrids is in question, as it has also been proposed that *C. eurytheme, C. eriphyle*, phenotypic intermediates, and other closely related entities (or some combination of these) might be polymorphic individuals of a single species (Edwards 1887; Gerould 1914; Shapiro 2012). As discussed later, apparent hybrids do occur in populations of *C. eriphyle* in western Canada, where *C. eurytheme* is absent or occurs only as a rare “stray.”

Hybridization, further interbreeding between hybrid individuals, and backcrossing between hybrids and parental types have produced hybrid swarms in some Lepidoptera species (Collins 1984; Mercader et al. 2009). However, the *Colias* system does not appear to maintain high hybrid abundance relative to parent species. In the eastern United States, *C. eurytheme* hybridizes naturally with *C. philodice,* which (as noted previously) has historically been considered the same species as *C. eriphyle* (Hovanitz 1950). Despite an initial high abundance of putative hybrids when the two species’ ranges first began to overlap in the late 1920s and early 1930s, hybrid abundance equilibrated at ∼10% in mixed populations (Clark 1932; Gerould 1946; Clark and Clark 1951). Additionally, high levels of interspecific mating between *C. eurytheme* and *C. eriphyle* in Arizona were seen to produce intermediate phenotype frequencies of up to 46% (Taylor 1972), but have since decreased to 10% hybrid phenotype individuals (Jahner et al. 2012). Hovanitz (1943) is the only source of published quantitative data on hybridization between the two species in California until Jahner et al. (2012); Hovanitz treats *C. philodice* and *C. eriphyle* as two races of the same species and also found 10% hybrid phenotypes. Laboratory rearings have proven *C. eurytheme* and *C. philodice* can produce viable F1 hybrids, F2 hybrids, and backcrosses with both parental types (Ae 1959; J. Gerould 1943). In addition to prior studies on the phylogenetic relationships within *Colias* using mtDNA (Pollock et al. 1998; Wheat and Watt 2008), information is available on the barriers to hybridization and the endogenous and exogenous drivers of apparent hybridization rates between *C. eurytheme* and *C. philodice* or *C. eriphyle* (Grula & Taylor Jr, 1979; Grula, McChesney, & Taylor, 1980; Jahner et al. 2012; Papke, Kemp, & Rutowski, 2007; RL Rutowski, 1980; Silberglied & Taylor Jr, 1978). These regulating factors include female response to pheromone and visual signals, abundance of parental species, species emergence dates, and maximum/minimum environmental temperatures.

It has been hypothesized that the introgression in both *C. eurytheme* and *C. eriphyle* in the Sierra Nevada is low based on the low abundance of putative hybrids at Sierra Valley, California. This site experienced drought conditions in 1994 and alfalfa senesced when irrigation ceased due to a low water table. *C. eurytheme* populations plummeted, but did not go locally extinct; *C. eriphyle* did go locally extinct and did not recolonize until a year and a half later. During *C. eriphyle*’s absence, hybrid and backcrossed phenotypes were absent from Sierra Valley, suggesting a lack of *C. eriphyle* phenotypic (and genetic) introgression in the *C. eurytheme* population (Jahner et al. 2012).

**Figure 3:**
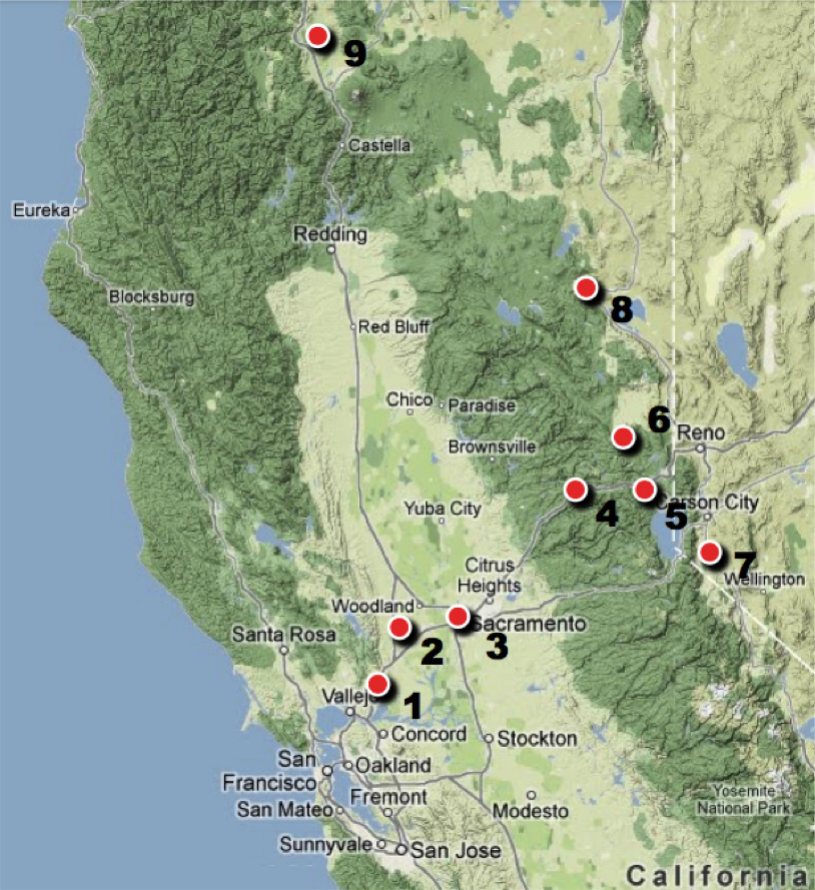
Collection sites. 1. Suisun, CA (38.23N, 122.04W). 2. Davis/Dixon, CA (38.44N, 121.86W). 3. Sacramento, CA (38.60N, 121.47W). 4. Lang Crossing, CA (39.32N, 120.66W). 5. Donner Pass, CA (39.32N, 120.37W). 6. Sierra Valley, CA (39.64N, 120.37W). 7. Gardnerville/Minden, NV (39. 00N, 119.80W). 8. Standish, CA (40.35N, 120.39W). 9. Montague, CA (41.76N, 122.51W). Sites 1, 2, and 3 are within the Sacramento Valley region; site 4 is within the west Sierra region; site 5 is within the High Sierra region; site 6 is within the East Sierra region; sites 7 and 8 are within the western Great Basin region, and site 9 is within the Shasta Valley region.

Samples of adult *C. eurytheme*, *C. eriphyle,* and phenotypic intermediates were collected from September 2011 to September 2012 from nine sites in northern California and Nevada that extend from the San Francisco Bay Area to the eastern slope of the Sierra Nevada and the vicinity of Mt. Shasta in the South Cascades (Fig.3). These sites can be divided into six regions: Sacramento Valley, West Sierra, High Sierra, East Sierra, western Great Basin, and Shasta Valley (Table1). *C. eurytheme, C. eriphyle,* and putative hybrids are present sympatrically in the latter three regions, and *C. eurytheme* occurs alone at the former three regions. Because *C. eriphyle* is absent in the Sacramento Valley, West Sierra, and High Sierra, all specimens collected from those regions were presumed to be “pure” *C. eurytheme*. At least fifteen specimens were collected from each region. Specimens were caught using a collapsible insect net, killed immediately, stored in glassine envelopes, and frozen within a few hours of capture.

**Table 1:** Samples used in genetic analyses by region.

Region	Number of *Colias eurytheme* individuals	Number of *Colias eriphyle* individuals	Number of phenotypic intermediates
Sacramento Valley	113	0	0
West Sierra	33	0	0
High Sierra	29	0	0
East Sierra	25	1	0
Western Great Basin	49	40	7
Shasta Valley	24	30	12

### DNA extraction and AFLP analysis

DNA was extracted from the head tissue of each sample using the protocol of the Qiagen DNeasy Blood and Tissue Kit (Germantown, MD, USA). AFLP analysis followed the methods of Vos et al. (1995), which involves digesting genomic DNA with two restriction enzymes (EcoRI and MseI), annealing specific primers to the ends of the restriction fragments, and reducing the number of restriction fragments through two rounds of selective polymerase chain reaction. Minor modifications to the procedure were implemented as described by Okada et al. (2009).

AFLP amplicon data were collected on an ABI 3130XL Capillary Electrophoresis Genetic Analyzer (Applied Biosystems, Foster City, CA, United States) and compiled for each primer combination with ABI GeneMapper software version 3.7 (Life Technologies, Carlsbad, CA, United States). GeneScan 500 ROX was used as an internal size standard. To minimize scoring errors, the threshold detection settings were adjusted so that fragment sizes <50 bp, higher than 500 bp, and lower than 100 reflective fluorescent units were not scored during the automated scoring process. All samples were evaluated manually for quality control following the automated scoring process. Any sample runs showing questionable size standards or peak signals were either scored by hand or omitted from the study. Only two individuals did not produce any clear genetic signal, and those individuals were omitted from the study. To confirm repeatability, a random sample of twenty individuals were run through the restriction, ligation, preselective PCR, selective PCR, fragment analysis, and scoring process an additional time. Any alleles that could not be accurately scored in both replicates (either automatically or by hand) were omitted. The final scored dataset consisted of 363 individuals from nine sites spanning six regions and 114 loci (Table1).

### Statistical analysis of population structure

The fragment presence/absence data were analyzed with STRUCTURE 2.3.4 (Pritchard et al. 2000), a software package which uses a Bayesian/Markov chain Monte Carlo method to probabilistically assign individuals to population genetic clusters based on genetic data only. The number of clusters (*K*) is identified based on the presence/absence data of all AFLP loci of all the individuals. Two possible models can be used to infer populations: either assuming no admixture or assuming admixture. Under the model that assumes no admixture, each individual is assumed to come purely from one of a number of discrete populations, and the posterior probability of each individual originating from a particular population is estimated. Under the model that assumes admixture and allows individuals to be of mixed ancestry, each individual’s genotype is proportionally assigned to the inferred populations. Because AFLP data consist of presence or absence of particular gene fragments, the methods of Falush et al. (2007) were followed to account for genetic ambiguity by defining fragment absences as the recessive state.

Before using STRUCTURE to analyze admixture between *C. eurytheme* and *C. eriphyle,* the no-admixture model was first used to determine whether individuals would cluster by species based on the AFLP data. This model included unambiguously phenotypic *C. eriphyle* individuals, and *C. eurytheme* individuals from the Sacramento Valley region where they do not occur in sympatry with *C. eriphyle* and have little chance of interbreeding with any stray *C. eriphyle* migrants.

Following the no-admixture species differentiation model run, the model assuming admixture was run using the genotypes of all individuals in order to address our first two research questions. Predesignated population information was included in the input file, but not incorporated into the model (i.e., the USEPOPINFO model was *not* incorporated). In this model, the results of the genetic clustering of individuals were visualized in three ways:

1. Species comparison, in which individuals were compiled into one of four groups: *C. eurytheme* from regions where *C. eriphyle* does not occur, *C. eurytheme* from regions where *C. eriphyle* occurs sympatrically, *C. eriphyle* from regions where *C. eurytheme* co-occurs*,* as all *C. eriphyle* collection sites were sympatric for the two species, and unambiguous phenotypic intermediates. In this comparison, assignment probabilities, especially those of sympatric *C. eurytheme* and intermediates, help indicate the extent of hybridization occurring between the two species.
2. Regional comparison, in which individuals were partitioned into one of the six collection regions (Sacramento Valley, West Sierra, High Sierra, East Sierra, western Great Basin, and Shasta Valley) and any morphological intermediates were *lumped* with *C. eurytheme*. This is because *C. eurytheme* are variable in the extent of orange coloration, while *C. eriphyle* are less so: They have constant, predictable light yellow coloration. So, while it might be reasonable to categorize *C. eriphyle* separately from any hybrid or backcrossed individuals, it is often difficult to detect the difference between a “pure” *C. eurytheme* individual and one that might be a *C. eurytheme* backcrossed hybrid.
3. Regional comparison, in which individuals were partitioned into one of the six collection regions and any individuals that unambiguously looked like hybrids were *segregated*. This comparison allows both the detection of the extent of hybridization in presumed hybrids and any differences in hybridization among different geographic regions.

Finally, we addressed our third research question by performing a comparison assuming admixture between sites only where phenotypic intermediates were caught (western Great Basin: Gardnerville/Minden, NV and Standish, CA; Shasta Valley: Montague, CA). Montague and the nearby collection site Gazelle have historically shown higher frequencies of phenotypic intermediates than other collection sites (i.e., approximately 25 vs. 10–15%). Using R software (R Development Core Team, 2012), unpaired and paired Welch *t*-tests were used to determine whether Montague and Gazelle (both located in Siskiyou Co., CA) each show significantly higher frequencies of intermediate phenotype individuals than Sierra Valley, CA. Unpaired *t*-tests incorporated annual mean hybrid frequencies from all sampling years we have on record; paired *t*-tests incorporated only mean hybrid frequencies from years where data exist for both sites in the comparison. The admixture model was then run in STRUCTURE to compare the extent of hybridization between *C. eurytheme* and *C. eriphyle* present at Montague, Standish, and Gardnerville/Minden. The population assignment probabilities were compared for intermediates collected at Montague vs. Standish and Gardnerville/Minden.

In all STRUCTURE runs, the model simulated 100 000 generations following an initial burn-in of 20 000 generations with 10 independent replicates of each *K* (between 1 and 6), where *K* is the number of potential population clusters. Longer runs of 1 000 000 generations did not significantly affect the assignment probabilities. The protocol of Evanno et al. (2005) was followed to infer the number of population clusters (Δ*K*) for the entire group of individuals. This method calculates Δ*K* based on the rate of change in the logarithm of the likelihood function of successive *K* values.

## Results

Four unique primer pairs generated a total of 114 AFLP bands ranging in size from 58 to 377 bp, 95.6% of which were polymorphic. The different primer pairs produced between 19 and 39 AFLP bands.

### Species differentiation: no-admixture model

**Figure 4:**
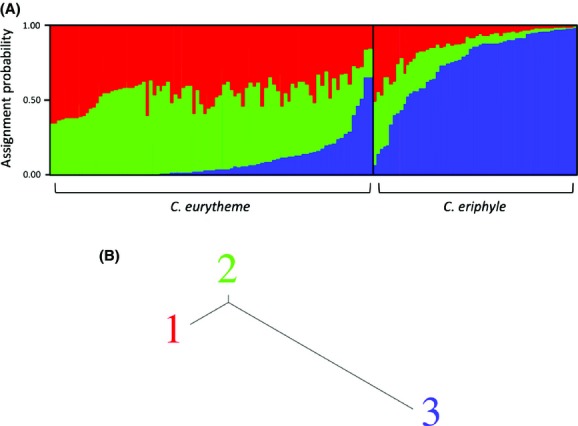
(A) Species differentiation bar plot assuming no admixture. Bayesian assignment probabilities of 142 individuals to *K* = 3 clusters. Each column corresponds to one individual, with color indicating cluster assignment probability. *Colias eurytheme* samples are from Sacramento Valley region and *Colias eriphyle* individuals are from western Great Basin and Shasta Valley regions (designated a priori using phenotype). Red = cluster 1, green = cluster 2, blue = cluster 3. (B) Neighbor-joining tree for species comparison data (assuming no admixture) representing the genetic distance among the *K* STRUCTURE clusters.

In the no-admixture model used to determine the presence of clustering between *C. eurytheme* and *C. eriphyle,* three clusters best explained the AFLP data. In this model, each individual’s assignment probability to a cluster estimates the proportion of the individual’s genome that originated from each cluster (Fig.4A). As averaged over 86 individuals, *C. eurytheme* were more likely to be assigned to clusters 1 or 2 (0.445 probability and 0.456 probability, respectively) than to cluster 3 (0.099 probability). As averaged over 55 individuals, *C. eriphyle* were more likely to be assigned to cluster 3 (0.732 probability) than to clusters 1 and 2 (0.134 probability for both). Even so, there was a wide range in the assignment probabilities to the three clusters among individuals within each species: in *C. eurytheme,* the probability of assignment to cluster 1 ranged between 0.159 and 0.644 and to cluster 2 ranged between 0.177 and 0.615; in *C. eriphyle,* the probabilities of assignment to cluster 3 ranged between 0.081 and 0.986. The unevenness in assignment probabilities between the two species (i.e., the lower proportion of assignment of *C. eurytheme* to cluster 3 and the relatively higher proportion of assignment of *C. eriphyle* to clusters 1 and 2) suggests that there is greater introgression in *C. eriphyle* than in *C. eurytheme*. The genetic distance (i.e., the net nucleotide distance that was calculated by applying a neighbor-joining algorithm to the matrix of allele-frequency divergence among clusters) between clusters 1 and 2 is 0.0005, between clusters 1 and 3 is 0.0035, and between clusters 2 and 3 is 0.0028, indicating that cluster 3 is more genetically distant than clusters 1 and 2 are to each other (Fig.4B).

### Species comparison and two regional comparisons: admixture model

**Figure 5:**
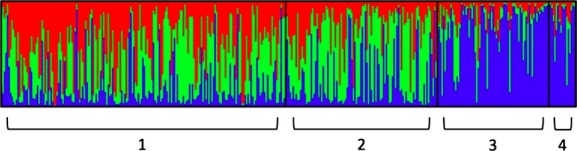
Species comparison bar plot assuming admixture. Bayesian assignment probabilities of 363 individuals to *K* = 3 clusters. Four groups were predefined for species comparison: 1 = *Colias eurytheme* from regions where *Colias eriphyle* is not present (i.e., Sacramento Valley, West Sierra, and High Sierra). 2 = *C. eurytheme* from regions where *C. eriphyle* is also present (i.e., East Sierra, western Great Basin, and Shasta Valley). 3 = *C. eriphyle*. 4 = phenotypic intermediates.

This analysis addressed our first question of whether there is genetic evidence for introgression between *C. eurytheme* and *C. eriphyle*. In the admixture-assuming model, four groups were predefined first for a species comparison: (1) *C. eurytheme* from regions where *C. eriphyle* does not occur (*“C. eurytheme* allopatric”), (2) *C. eurytheme* from regions where *C. eriphyle* co-occurs (“*C. eurytheme* sympatric”), (3) *C. eriphyle,* and (4) unambiguous phenotypic intermediates. Three clusters (*K* = 3) provided the best fit for the AFLP data. Of the individuals morphologically identified as *C. eurytheme,* there was relatively higher assignment of individuals to clusters 1 and 2 (Table2). In the *C. eurytheme* allopatric population, the average probability of assignment to cluster 1 was 0.418 and cluster 2 was 0.388, where most individuals’ genome was comprised of a significant proportion of each of the two clusters. In the *C. eurytheme* sympatric population, the average probability of assignment to the first two clusters was less even: assignment to cluster 1 was 0.258 and cluster 2 was 0.473. Of the individuals previously identified as *C. eriphyle* or phenotypic intermediates, there was relatively higher assignment of individuals to cluster 3. Of the *C. eriphyle* individuals, the average probability of assignment to cluster 3 was 0.727; and of the phenotypic intermediates, the average probability of assignment to cluster 3 was 0.791. These data indicate slightly higher levels of admixture in the *C. eurytheme* sympatric population than in the *C. eurytheme* allopatric population. Also, phenotypic intermediates appear to be genetically indistinguishable from *C. eriphyle* according to this model (Fig.5).

**Table 2:** Species comparison, “bulk groups”: proportion of membership of each predefined population in each of three clusters (*K* = 3).

Population	Inferred clusters		
	1	2	3
1. *Colias eurytheme* allopatric (Sacramento Valley, West Sierra, High Sierra)	0.418	0.388	0.194
2. *C. eurytheme* sympatric (East Sierra, western Great Basin, Shasta Valley)	0.258	0.473	0.269
3. *Colias eriphyle* (East Sierra, western Great Basin, Shasta Valley)	0.113	0.160	0.727
4. Phenotypic intermediates (East Sierra, western Great Basin, Shasta Valley)	0.100	0.109	0.791

**Figure 6:**
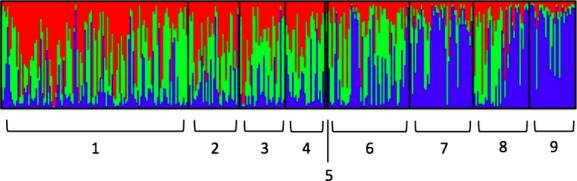
Regional comparison bar plot assuming admixture; intermediates lumped with *Colias eurytheme*. Bayesian assignment probabilities of 363 individuals to *K* = 3 clusters. Individuals are grouped by region of origin, and species assignments were designated a priori using phenotype. Phenotypic intermediates are lumped with *C. eurytheme* individuals of the same region. Nine groups were predefined for regional comparison: 1 = Sacramento Valley *C. eurytheme*. 2 = West Sierra *C. eurytheme*. 3 = High Sierra *C. eurytheme*. 4 = East Sierra *C. eurytheme*. 5 = East Sierra *Colias eriphyle*. 6 = Western Great Basin *C. eurytheme* and phenotypic intermediates. 7 = Western Great Basin *C. eriphyle*. 8 = Shasta Valley *C. eurytheme* and phenotypic intermediates. 9 = Shasta Valley *C. eriphyle*.

The results of the same admixture-assuming model were then visualized through a regional comparison. First, individuals were grouped by region and intermediates were lumped with *C. eurytheme* to avoid any erroneous categorization of mixed ancestry individuals, such that nine groups were predefined in this comparison: (1) Sacramento Valley *C. eurytheme,* (2) west Sierra *C. eurytheme,* (3) High Sierra *C. eurytheme,* (4) East Sierra *C. eurytheme,* (5) East Sierra *C. eriphyle,* (6) western Great Basin *C. eurytheme + *phenotypic intermediates, (7) western Great Basin *C. eriphyle,* (8) Shasta Valley *C. eurytheme + *phenotypic intermediates, and (9) Shasta Valley *C. eriphyle*. The proportions of assignment probability of each population to the three clusters reflect their geographic location (Fig.6). In the population of *C. eurytheme* individuals west of the Sierran crest, out of the three clusters, cluster 1 had the highest probability of assignment, while cluster 2 had the highest probability of assignment in *C. eurytheme* populations east of the Sierran crest. In both *C. eriphyle* populations and in the *C. eurytheme* (plus intermediates) in the Shasta Valley region, cluster 3 of the three clusters had the highest probability of assignment (Table3). There appears to be a gradual change in the genetic makeup of *C. eurytheme* from west to east, with increasing proportions assigned from cluster 1, to cluster 2, to cluster 3.

**Table 3:** Regional comparison, intermediates lumped with *Colias eurytheme:* proportion of membership of each predefined population in each of three clusters (*K* = 3).

Regional population	Inferred clusters		
	1	2	3
1. Sacramento Valley *C. eurytheme*	0.433	0.372	0.195
2. West Sierra *C. eurytheme*	0.409	0.383	0.208
3. High Sierra *C. eurytheme*	0.375	0.454	0.172
4. East Sierra *C. eurytheme*	0.309	0.469	0.222
5. East Sierra *Colias eriphyle*	0.120	0.160	0.720
6. Western Great Basin *C. eurytheme + *intermediates	0.209	0.450	0.341
7. Western Great Basin *C. eriphyle*	0.124	0.187	0.689
8. Shasta Valley *C. eurytheme + *intermediates	0.205	0.360	0.435
9. Shasta Valley *C. eriphyle*	0.071	0.124	0.805

**Figure 7:**
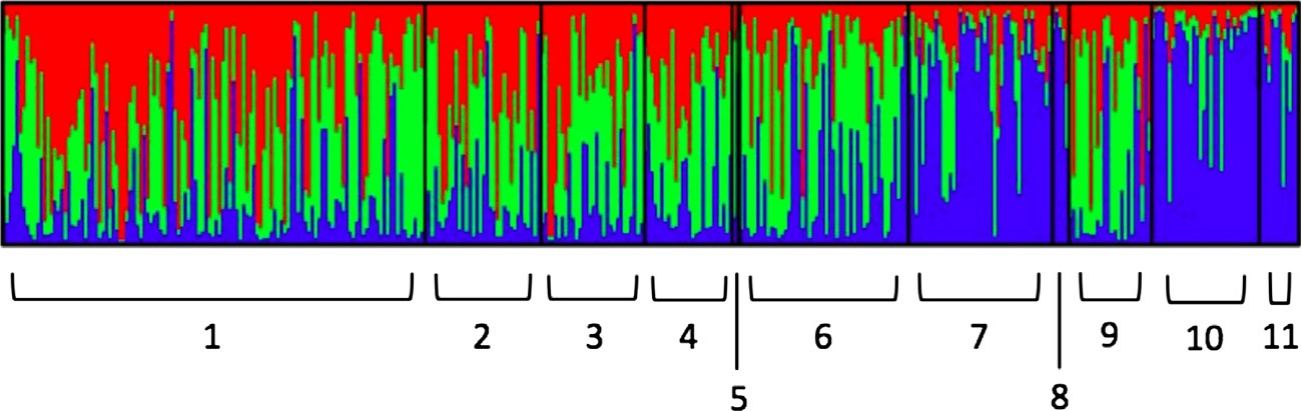
Regional comparison bar plot intermediates segregated from *Colias eurytheme*. Bayesian assignment probabilities of 363 individuals to *K* = 3 clusters. Individuals are grouped by regions of origin, and species assignments were designated a priori using phenotype. Unambiguous phenotypic intermediates are segregated from *C. eurytheme* individuals. Eleven groups were predefined for regional comparison: 1 = Sacramento Valley *C. eurytheme*. 2 = West Sierra *C. eurytheme*. 3 = High Sierra *C. eurytheme*. 4 = East Sierra *C. eurytheme*. 5 = East Sierra *Colias eriphyle*. 6 = Western Great Basin *C. eurytheme*. 7 = Western Great Basin *C. eriphyle*. 8 = Western Great Basin phenotypic intermediates. 9 = Shasta Valley *C. eurytheme*. 10 = Shasta Valley *C. eriphyle*. 11 = Shasta Valley phenotypic intermediates.

We performed a similar visual comparison to address our second question of whether there is genetic evidence that individuals of intermediate coloration are of mixed parentage. In this comparison, individuals were grouped by region in the same manner as before, except unambiguous phenotypic intermediates were segregated from *C. eurytheme,* such that eleven groups were predefined in this comparison*. *This allowed a comparison of presumed hybrids at a regional level. Genetically, phenotypic intermediates are indistinguishable from *C. eriphyle* (Fig.7). In the western Great Basin region, cluster 3 makes up a higher proportion of the cluster assignment probability in intermediates than in *C. eriphyle,* while the Shasta Valley region shows an opposite pattern: Cluster 3 makes up a higher proportion of the cluster assignment probability in *C. eriphyle* than in intermediates (Table4).

**Table 4:** Regional comparison, intermediates segregated from *Colias eurytheme:* proportion of membership of each predefined population in each of three clusters (*K* = 3).

Regional population	Inferred clusters		
	1	2	3
1. Sacramento Valley *C. eurytheme*	0.427	0.377	0.196
2. West Sierra *C. eurytheme*	0.399	0.393	0.208
3. High Sierra *C. eurytheme*	0.366	0.460	0.174
4. East Sierra *C. eurytheme*	0.313	0.465	0.221
5. East Sierra *Colias eriphyle*	0.113	0.175	0.713
6. Western Great Basin *C. eurytheme*	0.215	0.491	0.294
7. Western Great Basin *C. eriphyle*	0.128	0.190	0.682
8. Western Great Basin intermediates	0.119	0.076	0.805
9. Shasta Valley *C. eurytheme*	0.277	0.478	0.245
10. Shasta Valley *C. eriphyle*	0.073	0.124	0.803
11. Shasta Valley intermediates	0.087	0.124	0.789

### Site comparison: Gardnerville/Minden, Standish, and Montague

These results address our third research question of whether there is genetic evidence of higher levels of introgression at Montague, a site where high frequencies of phenotypic intermediates were historically observed. Analyses of historical data collected by one of us (from 1984 to 2000) revealed that Montague showed significantly higher annual frequencies of phenotypic hybrids than Sierra Valley for both paired and unpaired *t*-tests (*P* = 2.379e-08 and *P* = 3.651e-09, respectively). Similarly, the nearby town Gazelle (for which data were available from 1980 to 1998) showed significantly higher frequencies of phenotypic hybrids than Sierra Valley for both paired and unpaired *t*-tests (*P* = 2.147e-06 and *P* = 1.415e-08, respectively). Montague and Gazelle did not significantly differ from one another in their frequencies of phenotypic hybrids, which was expected, as the two sites are approximately 24 kilometers apart and could be considered the same *Colias* population. Similar data do not exist for other sites of record, so we were limited in our comparison of Montague to other sites, but these results show that Montague has a consistently higher frequency of phenotypic intermediates than at least one other collection site.

**Figure 8:**
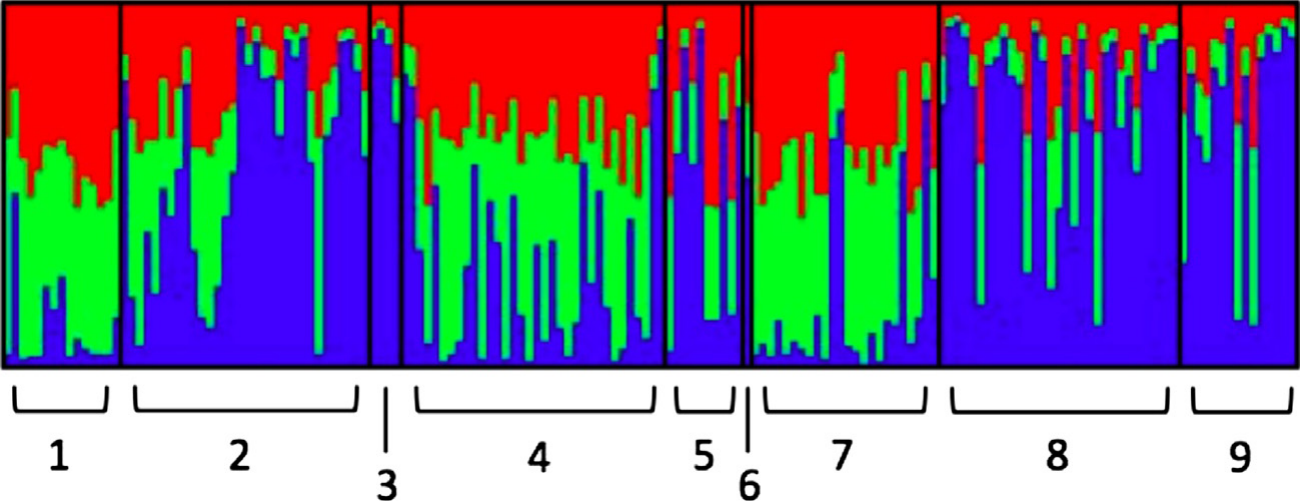
Site comparison bar plot: Gardnerville/Minden, Standish, and Montague. Bayesian assignment probabilities of 166 individuals to *K* = 3 clusters. Individuals are grouped by sites of origin, and species assignments were designated a priori using phenotype. 1 = Gardnerville/Minden *Colias eurytheme*. 2 = Gardnerville/Minden *Colias eriphyle*. 3 = Gardnerville/Minden phenotypic intermediates. 4 = Standish *C. eurytheme*. 5 = Standish *C. eriphyle*. 6 = Standish phenotypic intermediates. 7 = Montague *C. eurytheme*. 8 = Montague *C. eriphyle*. 9 = Montague phenotypic intermediates.

The AFLP site comparison analysis for Gardnerville/Minden, Standish, and Montague resulted in three clusters (*K* = 3) again providing the best fit for the data. For all three sites, the genetic makeup of *C. eurytheme* individuals was largely assigned to a mixture of clusters 1 and 2 (Fig.8). There does not appear to be a regional trend regarding the proportion of assignment to cluster 3 in *C. eurytheme* individuals (Table5). There does, however, appear to be a regional trend in the genetic makeup of *C. eriphyle:* on average, *C. eriphyle* individuals present at Shasta Valley site Montague are assigned a higher proportion to genetic cluster 3 (0.732) than *C. eriphyle* at western Great Basin sites Gardnerville/Minden and Standish (0.581 and 0.485, respectively). In phenotypic intermediates at all three sites, on average more than half of the genome is assigned to cluster 3, but there does not appear to be a regional trend. The genetic assignment proportions in intermediates from Standish and Montague are similar to proportions assigned in their *C. eriphyle* counterparts, but the proportions in intermediates from Gardnerville/Minden are quite different from *C. eriphyle* from Gardnerville/Minden (Table5).

**Table 5:** Site comparison: proportion of membership of each predefined population in each of three clusters (*K* = 3).

Regional population	Inferred clusters		
	1	2	3
1. Gardnerville/Minden *Colias eurytheme*	0.427	0.468	0.104
2. Gardnerville/Minden *Colias eriphyle*	0.211	0.208	0.581
3. Gardnerville/Minden intermediates	0.054	0.086	0.860
4. Standish *C. eurytheme*	0.413	0.301	0.286
5. Standish *C. eriphyle*	0.148	0.367	0.485
6. Standish intermediates	0.172	0.321	0.507
7. Montague *C. eurytheme*	0.411	0.418	0.171
8. Montague *C. eriphyle*	0.131	0.136	0.732
9. Montague intermediates	0.179	0.123	0.698

## Discussion

### Overall population structure

In all analyses, three genetic clusters (*K* = 3) provide the best explanation for the data. *C. eurytheme* individuals appear to be assigned mostly to a combination of two clusters (represented as red and green in the bar plots) and *C. eriphyle* individuals appear to be assigned mostly to a third cluster (represented as blue in the bar plots). According to the neighbor-joining tree, clusters 1 and 2 are more genetically similar to one another than either are to cluster 3, which supports the current understanding of *C. eriphyle* as a distinct species from *C. eurytheme*, although the high variation in the proportions of cluster assignments within species indicates either very recent species divergence or significant interbreeding. Within *C. eriphyle* individuals, the average genome proportion assigned to cluster 2 is usually equal to or higher than the proportion assigned to cluster 1. Moreover, in *C. eurytheme* individuals from regions where both species are sympatric, the average genome proportion assigned to cluster 2 is consistently higher than cluster 1. As the two species are sympatric only east of the Sierran Crest and *C. eurytheme* occurs alone west of the Sierran Crest, this implies a geographic pattern to clusters 1 and 2, where cluster 1 is more prevalent west of the Sierran crest and cluster 2 is more prevalent east of the Sierran Crest. The two-cluster genetic makeup of *C. eurytheme* might suggest that (1) this species is a mix of two closely related populations, which potentially were originally geographically separate, but which over time have become more mixed via continual gene flow, leading to the patterns we see today, or (2) this species is beginning to diverge into two distinct populations, potentially due to geographic isolation.

### Evidence for hybridization between *C. eurytheme* and *C. eriphyle*

According to the AFLP data, there is hybridization between the two species. In all models and levels of comparison, a significant proportion of the genetic makeup of *C. eurytheme* individuals assigned to cluster 3. In *C. eurytheme* populations where *C. eriphyle* does not co-occur, the average proportion assigned to cluster 3 is 0.194, while it is 0.269 in regions where *C. eriphyle* does co-occur. This suggests higher levels of hybridization where the two species are sympatric, as would be expected; evidence of hybridization west of the Sierra, where only *C. eurytheme* occurs, would reflect either historic gene flow or very low-level introgression across the Sierra Nevada. In *C. eriphyle,* proportion of assignment to clusters 1 and 2 combined is 0.273, which implies that the extent of introgression between the two species is fairly equal.

When phenotypic intermediates are lumped with *C. eurytheme,* the proportion of assignment to cluster 3 in this group is higher than in *C. eurytheme* when intermediates are excluded. As it is sometimes difficult to confidently identify the parentage of an individual based on phenotype (i.e., a backcrossed *C. eurytheme* specimen may be hard to distinguish from a pure *C. eurytheme* specimen), it is worthwhile to consider the genetic patterns when phenotypic intermediates are lumped with *C. eurytheme*. This avoids ambiguity when assigning predetermined populations in the STRUCTURE analysis. However, considering the genetic makeup of the phenotypic intermediates (as discussed below), it is likely that phenotypic intermediates are indeed genetically distinct from *C. eurytheme* and thus they should be considered separate entities when considering the extent of hybridization between *C. eurytheme* and *C. eriphyle*.

At the site level, there appear to be unusually high levels of introgression in *C. eriphyle* at Standish, CA. The individual genomes in this population are assigned on average 14.8% to cluster 1, 36.7% to cluster 2, and 48.5% to cluster 3. However, this pattern may be an artifact of a small sample size, as only ten *C. eriphyle* specimens were collected from Standish.

### Evidence that individuals of intermediate coloration are not true hybrids

Surprisingly, it does not appear that individuals of intermediate coloration (which have long been assumed to be hybrids) are of mixed parentage. At every comparison level (species, region, and site), phenotypic intermediates are genetically indistinguishable from *C. eriphyle,* as the majority of individual genetic assignments in both of these groups are to genetic cluster 3. In the western Great Basin region, the intermediates have even higher probabilities of assignment to cluster 3 than do *C. eriphyle* individuals. These data suggest that what were assumed to be hybrids are instead color morphs of *C. eriphyle*.

There are several lines of evidence, in addition to the genetic data, that support this conclusion. First, these phenotypic intermediates occur in sympatry with *C. eriphyle* wherever *C. eriphyle* occurs, including in western Canada where *C. eurytheme* is very rare or absent (N. Kondla, C. Guppy, personal communication to AMS). If phenotypic intermediates were true genetic hybrids, individuals with distinct orange coloration should not be observed in regions where *C. eurytheme* is rare or absent. Second, when *C. eriphyle* went temporarily extinct in Sierra Valley in 1994 due to drought and alfalfa senescence, intermediate phenotypes were also absent from that site until *C. eriphyle*’s re-colonization a year and a half later. If these intermediate phenotypes were true genetic hybrids, one would not expect all phenotypic hybrids and backcrosses to disappear along with *C. eriphyle* (Jahner et al. 2012). Third, it has been shown that laboratory-reared female hybrids resemble either their *C. eriphyle* or *C. eurytheme* parent as expected by X-chromosome supergenes (Grula and Taylor 1980), which would suggest that intermediate phenotype females would not exist in the wild. However, intermediate phenotype females have indeed been observed in the wild, albeit at lower frequencies than male intermediate phenotypes (Jahner et al. 2012). If these phenotypic intermediates were instead color morphs of *C. eriphyle,* then their phenotypes would not be a result of parental X-chromosome supergene inheritance and would explain the observation of female phenotypic intermediates in the wild. Other systems exist in which individuals that look like hybrids have been found to instead be phenotypic variations (Wysocka et al. 2011), and it has been shown that morphological phenotype does not necessarily correspond to introgressive hybridization (Harper and Hart 2007).

### Introgression levels at a site where high frequencies of phenotypic intermediates have been historically observed

The extent of introgression at Montague, a site that historically shows unusually high frequencies of phenotypic hybrids, does not appear to be any higher than at other sites where both species occur. Standish, a western Great Basin site, shows stronger evidence for introgression, as Standish *C. eriphyle* individuals exhibited higher probabilities of assignment to cluster 2 than *C. eriphyle* at other sites. Although this result is unexpected if one assumes the high frequency of phenotypic intermediates is indeed a high frequency of genetic hybrids, it is less surprising if one considers that phenotypic intermediates are in fact genetically indistinguishable from *C. eriphyle*. If the phenotypic intermediates are indeed a color morph of *C. eriphyle*, there is no reason why one would expect higher levels of introgression at Montague. The frequency of different color morphs within *C. eriphyle* would be determined by their fitnesses, not the frequency of interspecific pairings.

## Conclusion

From this genetic analysis, we conclude that the *Colias eurytheme* and *Colias eriphyle* complex is made up of three genetic clusters, with individuals of *C. eurytheme* primarily assigned to two closely related clusters and *C. eriphyle* mostly assigned to a third, more distantly related cluster. As expected, there appears to be significant hybridization between the two species. However, individuals of intermediate phenotype, which have historically been considered hybrids of the two species, were found to be genetically indistinguishable from *C. eriphyle*. Given the relatively low sampling of phenotypic intermediates, some caution is warranted in the interpretation of these results. Nevertheless, our conclusion indicates that previous work based on the assumption that these intermediate forms are hybrids may warrant reconsideration.

Further molecular work such as gene and genome sequence data analysis would strengthen these conclusions. Future research would benefit from sampling *C. eriphyle* from locations where *C. eurytheme* is extremely rare or absent, which would help clarify the population structure of this species complex. Additionally, a morphological comparison of laboratory-reared hybrids and wild-caught phenotypic intermediates could elucidate any subtle differences between true hybrids and hybrid-looking *C. eriphyle*. Because such differences are not evident, and because of the documented history of recently sympatric populations in eastern North America and in Arizona (initially high levels of phenotypic intermediacy, followed by rapid stabilization around 10%), it is possible that “orange” genes from *C. eurytheme* have repeatedly entered “yellow” populations via hybridization and, at least in the case of *C. eriphyle,* introgressed widely, becoming a functional color polymorphism even in allopatric populations, extending far beyond any obvious genomic signature of hybridization. Testing this hypothesis will require identification and sequencing of the genes determining orange coloration in both *C. eurytheme* and phenotypic intermediates.
